# Outcomes in patients with acute upper gastrointestinal bleeding following changes to management protocols at an Australian hospital

**DOI:** 10.1002/jgh3.12303

**Published:** 2020-02-05

**Authors:** Adrianna Papadinas, Joshua Butt

**Affiliations:** ^1^ Department of Gastroenterology Northern Health, Epping Melbourne Victoria Australia

**Keywords:** acute, endoscopy, management, outcomes, protocols, risk stratification, transfusion, upper gastrointestinal bleeding

## Abstract

**Background and Aim:**

Upper gastrointestinal bleeding (UGIB) has a high mortality rate and requires efficient and directed acute management. This project aimed to assess patient outcomes following changes to UGIB management protocols at Northern Hospital, Victoria, Australia. Changes involved streamlining management under a single inpatient unit, earlier endoscopy, blood transfusion thresholds, and risk stratification.

**Methods:**

This was a cohort study of 400 patients aged ≥18 years admitted to Northern Hospital who underwent endoscopy for acute UGIB. Data of preprotocol changes (Group 1) and prospectively postprotocol changes (Group 2) were collected retrospectively. Primary outcomes were inpatient mortality, rebleeding, radiologic or surgical intervention, and endoscopic reintervention. Secondary outcomes included length of stay (LOS) ≥4 days and blood units transfused. Univariate analyses were conducted comparing groups and associations between variables and outcomes, followed by multivariate analyses for each outcome.

**Results:**

There was no difference in mortality on multivariate analysis (*P* = 0.95). Rebleeding reduced by 4% (adjusted odds ratio [AOR] 0.48; *P* = 0.03), LOS ≥4 days reduced by 15.1% (AOR 0.46; *P* < 0.00) and median blood units transfused decreased with adjusted incidence rate ratio of 0.81 (*P* = 0.00). Early endoscopy (i.e. ≤12 h) for all patients increased by 15% (*P* < 0.00) and there were 12% more high‐risk patients (i.e. Glasgow–Blatchford score ≥ 12) in Group 2 (*P* = 0.01).

**Conclusion:**

Following changes to UGIB protocols at this Australian hospital, endoscopic times decreased with reductions in rebleeding, LOS ≥4 days, and blood transfusion rates. These findings demonstrate improved outcomes after the implementation of new treatment targets focusing on streamlined care of patients presenting with UGIB.

## Graphical abstract

The 2017 upgrade of management protocols for acute upper gastrointestinal bleeding at Northern Hospital focused on faster times to endoscopy, reduced blood transfusion thresholds and new risk stratification tools. A review of patient outcomes following these changes demonstrated significant improvements in re‐bleeding rates, length of stay and blood transfusion rates.

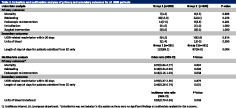

## Introduction

While the prevalence of upper gastrointestinal bleeding (UGIB) has decreased over previous decades, significant mortality rates remain despite advances in diagnostic and management tools.^1^ Initial management generally involves fluid resuscitation, hemodynamic monitoring and blood transfusions,^2^ which should occur prior to endoscopic intervention.^3^ The efficacy of UGIB management protocols can be evaluated by reviewing patient outcomes such as mortality and rebleeding. The 2017 Northern Health upgrade of acute UGIB management protocols outlined in a concise flowchart focused on streamlining patient care as well as changes to risk stratification and blood transfusion thresholds. Management of patient care under a single inpatient unit (Gastroenterology) within and after‐hours (i.e. evenings and weekends) replaced previous protocols, whereby care was shared between surgical and medical teams. This aimed to hasten referrals and reduce time to endoscopy to within timeframes from the 2010 International Consensus Guidelines^3^ and National Institute for Health and Clinical Excellence (NICE) 2012 guidelines.^4^ These timeframes are associated with improved outcomes although additional guidance based on specific risk factors is awaited.^5^ The Glasgow–Blatchford score (GBS) replaced the Rockall score to stratify high‐risk patients based on the superior efficiency of GBS in predicting the need for endoscopic intervention.^6^ Blood transfusion thresholds for asymptomatic patients were lowered to hemoglobin <70 g/L in the absence of hemodynamic instability despite resuscitation or concurrent acute coronary syndrome, in concordance with trials supportive of a “restrictive” approach.^7^ Overall, this study aimed to assess changes in UGIB patient outcomes after streamlining care to a single UGIB‐dedicated unit with the purpose of expediting endoscopy times in conjunction with other acute management upgrades. Improvements in outcomes would therefore support the ongoing need for specialized units to manage these high‐risk patients despite the overall reduced prevalence of UGIB.

## Methods

### *Study design and population*

This was a cohort study of 400 patients aged ≥18 years with acute UGIB admitted to the Northern Hospital who underwent endoscopy. Data from 200 patients in Group 1 were collected retrospectively prior to protocol changes between March 2016 and May 2017. Group 2 consisted of 200 patients whose data were collected retrospectively following protocol changes from May 2017 to March 2018. Multiple sources were used for data collection including the endoscopy reporting software at the Northern Hospital (Endobase), searches of hospital coding by Client Data Management, and from a prospectively collected UGIB audit initiated in the new protocols. Remaining data were collected from patient files accessed from the Central Processing Unit.

### *Patient characteristics*

Patients were categorized in subgroups of demographics, that is, age and gender, medication, comorbid illness, admission (i.e. from emergency department [ED] or inpatient), treating team, presentation (i.e. melena, hematemesis, mixed or other), preendoscopic assessment and treatment including systolic blood pressure (SBP), hemoglobin (Hb) and need for blood transfusion. Endoscopic diagnoses were categorized as no abnormalities detected, nonvariceal bleeding, and variceal bleeding. Continuous variables were dichotomized, that is, age (≥65 and <65 years), Hb (≥70 and <70 g/L), and SBP (≥100 and <100 mmHg). Comorbidities recorded included organ failure or malignancy and combinations of diseases were categorically quantified. Medications were recorded for drug classes with increased UGIB risk, including antithrombotic, antidepressants, and nonsteroidal anti‐inflammatory drugs (NSAIDS) or steroids.^8^ Antiplatelet medications included monotherapy or dual antiplatelet therapy which were compared between groups but not included in further analysis. Cessation of antithrombotic medications was not recorded.

### *Time to endoscopy*

Time to endoscopy was defined from initial referral by the treating team to the commencement of the procedure based on anesthetics records. Timeframes were measured as <6 h, 6–12 h, 12–24 h, and >24 h. They were further categorized as <12 h and <24 h for all patients, and <6 h for variceal UGIB patients. Specific timeframes analyzed were based on new protocols recommendations, including endoscopy within 24 h for nonvariceal UGIB patients with GBS ≥7, and early endoscopy (i.e. ≤12 h) for high‐risk nonvariceal patients i.e. GBS ≥12. Urgent endoscopy (i.e. <6 h) was indicated for active ongoing bleeding or variceal bleeding.

### *Risk stratification*

The Glasgow–Blatchford score (GBS) was calculated for each patient with low risk defined as GBS <12, and high risk as GBS ≥12. Blood pressure values used to calculate the GBS were from the initial presentation, generally postresuscitation for ED admissions or on initial assessment by the treating team for inpatient admissions.

### *Patient outcomes*

The primary outcomes were inpatient mortality, inpatient rebleeding, need for surgical or radiological intervention, and endoscopic reintervention. Rebleeding was defined as melena, hematemesis or other clinical concerns of rebleeding after initial endoscopy. Endoscopic reintervention was defined as repeat endoscopy for the purpose of achieving hemostasis of rebleeding. Secondary outcomes included length of stay (LOS) ≥4 days, readmission within 30 days, and units of blood transfused. LOS ≥4 days was selected as it was the lowest median value between groups. Patients presenting with UGIB as inpatients were excluded when analyzing LOS as ongoing treatment was generally non‐UGIB related. Units of blood transfused were recorded as the total number of packed red blood cells received after suspected or confirmed UGIB. Readmission within 30 days included UGIB‐related cases only. Intensive care unit (ICU) admissions were excluded in this study.

### *Data analysis*

Differences between groups were identified using Chi tests for discrete variables. Patient characteristics were summarized using absolute values and percentages for categorical variables, and with medians and interquartile ranges for continuous variables. Skewness was established by testing for normality with Shapiro–Wilk tests. Significance was defined as *P* value <0.050. Univariate analysis was then carried out comparing UGIB patients with nonvariceal *versus* variceal UGIB. Mann–Whitney U tests were used for continuous variables with binary factors and Kruskal–Wallis tests for multi‐factored continuous variables. Further univariate analysis identified significant associations between variables and each outcome. Multivariate analyses were conducted with generalized linear analyses using a forward stepwise selection process adjusting for variables found to significantly associated on univariate analysis, or those statistically different between groups. Only variables with a *P* value of <0.050 when adjusted for were included in the multivariate analysis. These variables included: admission (i.e. from ED or as inpatient), GBS, antiplatelet use, anticoagulant use, blood transfusion requirement, age, hemoglobin level and gender. The baseline adjusted odds ratios (AOR) and an adjusted incidence rate ratio (AIRR) of 1 was allocated to Group 1 for multivariate analyses. Univariate analysis was also used to compare patients with nonvariceal *versus* variceal UGIB for time to endoscopy and each outcome. The percentage of patients requiring endoscopic intervention based on GBS was calculated using the absolute values and percentages.

## Results

### *Patient characteristics*

Four hundred patients were included in the cohort with 200 patients (50%) in Group 1 preprotocol changes and 200 patients (50%) in Group 2 postprotocol changes. There was no significant difference in patient demographics between groups in terms of age, gender, medication usage (including use of dual antiplatelet therapy), presentation, or endoscopic diagnosis (Table 1). UGIB patients treated by the Gastroenterology unit increased by 63.5% as expected based on the new protocol (Table 1). Patients presenting with UGIB as inpatients increased significantly from 19 (9.5%) in Group 1 to 38 (19%) in Group 2 (*P* = 0.006) (Table 1). Regarding preendoscopic assessment and treatment, SBP and hemoglobin levels were not different between groups, while the requirement for blood transfusions of any amount increased insignificantly by 3% (Table 1).

**Table 1:** Characteristics of patients with acute upper gastrointestinal bleeding (UGIB) receiving endoscopy in Group 1 (preprotocol changes) *versus* Group 2 (postprotocol changes)

Characteristics	Group 1 (*n* = 200)	Group 2 (*n* = 200)	*P*‐value
Demographics			
Age ≥ 65	114 (57.0)	119 (59.5)	0.257
Sex (male)	148 (74.0)	131 (65.5)	0.064
Medication			
Antiplatelet therapy	82 (41.0)	89 (44.5)	0.479
Anticoagulation	33 (16.5)	30 (15.0)	0.680
Antidepressant	35 (17.5)	36 (18)	0.896
NSAIDS/steroids	29 (14.5)	32 (16)	0.676
Comorbidities			
No comorbidities	109 (54.4)	105 (52.5)	0.904
One comorbidity	72 (36.0)	74 (37.0)	—
Two or more comorbidities	19 (9.5)	21 (10.5)	—
Admission and treating team			
Inpatient admission	19 (9.5)	38 (19.0)	0.006
ED admission	181 (90.5)	162 (81.0)	—
Treating team			
Gastroenterology unit	51 (25.5)	178 (89.0)	<0.000
Presentation			
Melena	93 (46.5)	94 (47.0)	0.608
Hematemesis	45 (22.4)	50 (25.0)	—
Mixed	43 (21.5)	35 (17.5)	—
Other	18 (9.0)	21 (10.5)	—
Preendoscopic assessment and treatment			
Systolic blood pressure < 100 mmHg	25 (12.5)	25 (12.5)	1.000
Hb < 70	27 (13.5)	37 (18.5)	0.172
Blood transfusion required	113 (56.5)	119 (59.5)	0.543
Risk stratification scores			
Glasgow‐Blatchford score ≥ 12	48 (24.0)	72 (36.0)	0.009
Endoscopy			
Endoscopic diagnosis			
No abnormalities detected	36 (18.0)	32 (16.0)	0.687
Nonvariceal	142 (71.0)	141 (70.5)	—
Variceal	22 (11.0)	27 (13.5)	—
Time to endoscopy for all UGIB patients			
≤24 h	146 (73.0)	173 (86.5)	0.001
≤12 h	92 (46.0)	122 (61.0)	0.003

Proportions presented as absolute value (%). Median and interquartile range as median [25th percentile, 75th percentile]. Notation used throughout.

CI, confidence interval; ED, emergency departemnt; Hb, hemoglobin; NSAIDS, nonsteroidal anti‐inflammatory drugs.

### *Risk stratification*

**Figure 1:**
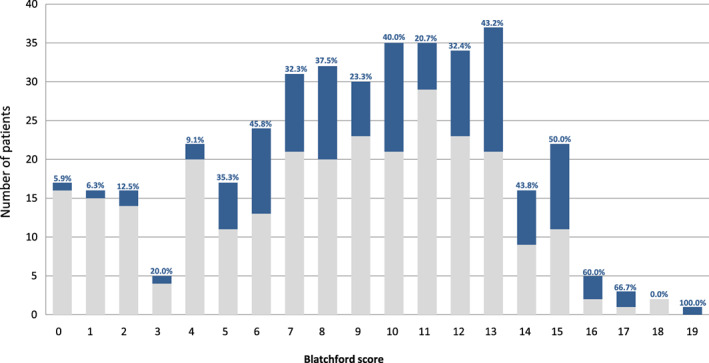
Percentage of patients requiring endoscopic intervention based on Glasgow–Blatchford score (GBS). By comparing the number of patients who required intervention *versus* those who did not an association between an increasing GBS (i.e. increasing risk from 0–19) and need for intervention is demonstrated. (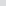), No endoscopic intervention; (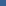), endoscopic intervention.

There was a 12% increase in patients with high‐risk GBS of ≥12 in Group 2 (*P* = 0.009). Regarding the association between GBS score and need for endoscopic intervention, a larger percentage of intervention was required in patients with higher GBS (Fig. 1). Of the 17 patients with a GBS of 0, 5.9% received endoscopic intervention. The average amount of endoscopic intervention for low‐risk GBS was 24.1 and 49.5% for those with high‐risk GBS.

### *Time to endoscopy*

The overall number of patients receiving endoscopy within 24 h increased from 73% in Group 1 to 86.5% in Group 2, while the number of patients receiving early endoscopy (i.e. ≤12 h) increased by 15% (Table 1). When patients were classified according to their endoscopic diagnosis for UGIB (i.e. nonvariceal *vs* variceal) the improvements in time to endoscopy for both the 24 and 12 h timeframes remained significant for the nonvariceal group (Table 3). The number of nonvariceal patients with GBS ≥7 receiving endoscopy within 24 h increased significantly by 13.2%, though the number of nonvariceal patients with GBS ≥12 receiving early endoscopy did not significantly improve with an 8.6% increase (Table 3). There were no improvements in time to endoscopy for the variceal group of patients with the number of patients receiving endoscopy within 24 h decreasing from 100.0 to 88.9%, and from 77.3 to 74.1% within 12 h (Table 3). Notably there was a significant drop for variceal patients receiving urgent endoscopy (i.e. within 6 h) from 72.7% in Group 1 to 44.4% in Group 2 (Table 3).

### *Primary outcomes*

Seven patients died (3.5%) from Group 1 and nine (4.5%) from Group 2 (*P* = 0.609) on multivariate analysis (AOR 0.97; 95% confidence interval (CI) 0.34–2.77; *P* = 0.952) (Table 2). Rebleeding reduced by 4%, which was significant on multivariate analysis (AOR 0.48; 95% CI 0.25–0.92, *P* = 0.026) (Table 2). Endoscopic reintervention dropped from 7 to 4.5% which bordered on significance on multivariate analysis (AOR 0.41; 95% CI 0.16–1.03; *P* = 0.058) (Table 2) There were two cases requiring embolization in each group (*P* = 1.000) with no significant findings between groups (Table 2). There were no cases of surgical intervention.

**Table 2:** Univariate and multivariate analyses of primary and secondary outcomes for all upper gastrointestinal bleeding (UGIB) patients

Univariate analysis	Group 1 (*n* = 200)	Group 2 (*n* = 200)	*P*‐value
Primary outcomes			
Mortality	7 (3.5)	9 (4.5)	0.609
Rebleeding	30 (15.0)	22 (11)	0.233
Endoscopic reintervention	14 (7.0)	9 (4.5)	0.281
Embolization	2 (1.0)	2 (1.0)	1.000
Surgical intervention	0 (0.0)	0 (0.0)	1.000
Secondary outcomes			
UGIB‐related readmission within 30 days	9 (4.5)	10 (5.0)	0.814
Units of blood	2 [1–4]	1 [0–3]	0.990
	Group 1 (*n* = 181)	Group 2 (*n* = 161)	
Length of stay ≥4 days for patients admitted from ED only	125 (69.1)	87 (54.0)	0.004

Multivariate analysis	Odds ratio (95% CI)	*P*‐value
Primary outcomes†		
Mortality	0.97 (0.34–2.77)	0.952
Rebleeding	0.48 (0.25–0.92)	0.026
Endoscopic reintervention	0.41 (0.16–1.03)	0.058
Secondary outcomes		
UGIB‐related readmission within 30 days	0.93 (0.37–2.36)	0.879
Length of stay ≥4 days for patients admitted from ED only	0.46 (0.28–0.74)	0.001
	Incidence rate ratio (95% CI)	*P*‐value
Units of blood transfused	0.81 (0.70–0.93)	0.003

† Embolization was not included in this section as there were no significant findings on multivariate analysis for this outcome.

CI, confidence interval; ED, emergency department.

### *Secondary outcomes*

There was no significant change in readmission rates within 30 days (Table 2). LOS ≥4 days for patients admitted via ED (i.e. excluding inpatient presentations) reduced from 69 to 54%, (Table 2). Median units of blood transfused reduced from 2 to 1 (Table 2). When outcomes were reviewed separately for nonvariceal *versus* variceal groups on univariate analysis LOS ≥4 days for nonvariceal patients decreased significantly by 17.2% (Table 3). Regarding antithrombotic use, significant associations were found for multiple outcomes, including reduced rebleeding with antiplatelet therapy (Table 4). Anticoagulation use was significantly associated with LOS ≥4 days (Table 4).

**Table 3:** Univariate analysis of time to endoscopy and primary and secondary outcomes for nonvariceal *versus* variceal upper gastrointestinal bleeding (UGIB) patients

Nonvariceal UGIB patients	Group 1 (*n* = 142)	Group 2 (*n* = 141)	*P*‐value
Time to endoscopy			
≤24 h	104 (73.2)	126 (89.4)	<0.000
≤12 h	65 (45.8)	91 (64.5)	0.001
Time to endoscopy for patients with GBS ≥ 7	Group 1 (n = 98)	Group 2 (n = 106)	
≤24 h	73 (74.5)	98 (87.7)	0.015
Time to endoscopy for patients with GBS ≥ 12	Group 1 (*n* = 33)	Group 2 (*n* = 52)	
≤12 h	20 (60.6)	36 (69.2)	0.415
Primary outcomes			
Mortality	4 (2.8)	7 (5.0)	0.347
Rebleeding	16 (11.3)	14 (9.9)	0.714
Endoscopic reintervention	8 (5.6)	6 (4.3)	0.592
Embolization	2 (1.4)	2 (1.4)	0.994
Surgical intervention	0 (0.0)	0 (0.0)	1.000
Secondary outcomes			
UGIB‐related readmission within 30 days	6 (4.2)	6 (4.3)	0.990
Units of blood	2 [1–4]	1 [0–3]	0.851
	Group 1 (*n* = 129)	Group 2 (*n* = 109)	
Length of stay ≥4 days for patients admitted from ED only	86 (66.7)	54 (49.5)	0.007

Variceal UGIB patients	Group 1 (*n* = 22)	Group 2 (*n* = 27)	*P*‐value
Time to endoscopy			
≤24 h	22 (100.0)	24 (88.9)	0.053
≤12 h	17 (77.3)	20 (74.1)	0.795
≤6 h	16 (72.7)	12 (44.4)	0.044
Primary outcomes			
Mortality	3 (13.6)	1 (3.7)	0.202
Rebleeding	6 (27.3)	2 (7.4)	0.059
Endoscopic reintervention	3 (13.6)	1 (3.7)	0.202
Embolization	0 (0.0)	0 (0.0)	—
Surgical intervention	0 (0.0)	0 (0.0)	—
Secondary outcomes			
UGIB‐related readmission within 30 days	1 (4.5)	1 (3.7)	0.883
Units of blood transfused	2 [0–5]	1 [0–3]	0.074
	Group 1 (*n* = 20)	Group 2 (*n* = 24)	
Length of stay ≥4 days for patients admitted from ED only	17 (85.0)	17 (70.8)	0.258

Proportions presented as absolute value (%). Median and interquartile range as median [25th percentile, 75th percentile].

CI, confidence interval; ED, emergency department; GBS, Glasgow‐Blatchford score.

**Table 4:** Multivariate analysis of primary and secondary outcomes for patients presenting with upper gastrointestinal bleeding (UGIB)

	Primary outcomes†						Secondary outcomes					
	Mortality		Rebleeding		Endoscopic reintervention		Readmission within 30 days for UGIB		Length of stay ≥4 days for ED admissions		Units of blood transfused	
	Odds ratio (95% CI)	*P*‐value	Odds ratio (95% CI)	*P*‐value	Odds ratio (95% CI)	*P*‐value	Odds ratio (95% CI)	*P*‐value	Odds ratio (95% CI)	*P*‐value	Incidence rate ratio (95% CI)	*P*‐value
Group 1	1	—	1	—	1	—	1	—	1	—	1	—
Group 2	0.97 (0.34–2.77)	0.952	0.48 (0.25–0.92)	0.026	0.41 (0.16–1.03)	0.058	0.93 (0.37–2.36)	0.879	0.46 (0.28–0.74)	0.001	0.81 (0.70–0.93)	0.003
Variables												
ED admission	1	—	1	—	1	—					1	—
Inpatient admission	6.90 (2.42–19.57)	<0.000	2.55 (1.22–5.36)	0.013	4.32 (1.68–11.15)	0.002					2.02 (1.72–2.37)	<0.000
Blatchford < 12			1	—	1	—					1	—
Blatchford ≥ 12			3.04 (1.59–5.82)	<0.000	3.07 (1.25–7.51)	0.014					2.06 (1.76–2.40)	<0.000
No antiplatelet			1	—								
Antiplatelet therapy			3.11 (1.65–5.88)	0.001								
No anticoagulation									1	—		
Anticoagulation									3.23 (1.28–8.14)	0.013		
No blood transfusion							1	—	1	—		
Transfusion required							0.25 (0.07–0.87)	0.029	3.53 (2.16–5.77)	<0.000		
Age < 65 years									1	—		
Age ≥ 65 years									1.69 (1.03–2.76)	0.039		
Hb ≥ 70											1	—
Hb < 70											2.28 (1.95–2.66)	<0.000
Gender: female												
Gender: male												

† Embolization was excluded from this table as no significant variables were associated with this outcome.

CI, confidence interval; ED, emergency department; Hb, hemoglobin.

## Discussion

This was a cohort study looking at outcomes in patients with acute UGIB admitted to the Northern Hospital before and after the latest upgrade to management protocols. Overall there was a significant reduction in time to endoscopy with improvements in multiple patient outcomes including rebleeding rates, LOS and units of blood transfused. There was a significant difference in the number of patients presenting with UGIB as inpatients, with 9.5% in Group 1 and 19% in Group 2 (*P* = 0.006). This discrepancy is noteworthy when considering how patient outcomes changed between groups as inpatient admissions are associated with worse outcomes for UGIB patients.^9^ This is demonstrated in the inclusion of this variable in multivariate analyses of multiple outcomes including mortality (odds ratio [OR] 6.90; 95% CI 2.42–19.57; *P* < 0.000) (Table 4). There were also a larger number of high‐risk nonvariceal patients (i.e. GBS ≥ 12) in Group 2 indicating the overall improvements in outcomes were potentially underestimated in this study. The streamlining of patient management under Gastroenterology was achieved for most patients (89%) (Table 1). These results were possibly underestimated with delayed documentation for patients requiring urgent endoscopic procedures, or ICU admissions which were not recorded in this study.

### *Risk stratification*

The Glasgow–Blatchford score has demonstrated efficacy in predicting patient outcomes including need for endoscopic intervention, need for blood transfusion and mortality.^10^ In this study of all the patients with GBS = 0, 5.9% required intervention despite studies demonstrating this score to be strongly correlated no need for endoscopic treatment.^6^ This may be due to the exclusion of patients not receiving endoscopy. Furthermore while higher risk patients had relatively larger amounts of endoscopic intervention (Fig. 1), sample sizes were small and analyses from larger randomized populations is warranted. When GBS score was dichotomized (i.e. <12 or ≥12), a significant association was found between GBS ≥ 12 with rebleeding, the need for endoscopic reintervention and units of blood transfused (Table 4).

### *Time to endoscopy*

Overall there were significantly reduced times to endoscopy after the implementation of new timeframe targets for nonvariceal UGIB patients and the streamlined care under a single UGIB unit. Despite the overall improvements 13.5% of patients failed to receive endoscopy within 24 h post protocol changes and 39% within 12 h (Table 1). When looking at nonvariceal UGIB patients whom which the new protocol timeframes specifically targeted, there were significant improvements within 24 h by 16.2 and 18.7% within 12 h (Table 3). And 87.7% of nonvariceal patients with a GBS ≥7 in Group 2 received an endoscopy within 24 h, which was a significant increase from 74.5% in Group 1 (*P* = 0.015). There was an increase of 8.7% of high risk (i.e. GBS ≥ 12) nonvariceal patients receiving endoscopy within 12 h though this was not significant (*P* = 0.415), and 30.8% did not reach the recommended target (Table 3). Variceal UGIB patients generally have worse outcomes compared to nonvariceal and require faster times to endoscopy.^11^ This study found the number of variceal bleeders receiving urgent endoscopy (<6 h) dropped significantly from 72.7 to 44.4% (*P* = 0.044). There was also a slight decrease for these patients within 12 h by 3.2%, and within 24 h from 100.0 to 88.9% (Table 3). These findings were unexpected as variceal UGIB protocols were unchanged. Delays may be due to enhanced resuscitation measures required prior to endoscopy or a limitation on the ability to prioritize variceal bleeders with the increase in nonvariceal patients receiving earlier endoscopy (Table 2). Despite the increase in times to endoscopy for variceal UGIB patients there were no significant changes in their outcomes (Table 3).

### *Primary outcomes*

There was no significant change in the inpatient mortality rates for UGIB patients on univariate or multivariate analyses (Table 2). This is despite findings that rebleeding rates significantly decreased by 4% (Table 2) which is associated with reduced mortality.^12^ Furthermore there was an overall reduction in time to endoscopy which also correlates with reduced mortality, specifically when looking at endoscopy within 24 h.^13^ These discrepancies may be due to a number of limitations in the study design including the limited power with 400 patients. The nonrandomized nature of the study could have caused confounders not recognized or adjusted for on multivariate analysis. Only patients who had an endoscopy were included meaning patients who died prior to endoscopy were not recorded. This may have limited the analysis of how preendoscopic assessment and management impacted on patient prognosis. The cessation and recommencement of antithrombotic drugs was not included in this study which may be an influential determinant of patient outcomes, particularly on rebleeding which was found to be significantly associated with antiplatelet use (Table 4). The management of UGIB patients on antithrombotic medication is an ongoing area of research with further studies required to develop specific guidelines; current recommendations are for determination of risk‐*versus*‐benefit in case‐by‐case evaluations.^4^

### *Secondary outcomes*

There was no significant difference in readmission rate within 30 days between groups on univariate (*P* = 0.814) or multivariate analysis (*P* = 0.879), however data on readmission at other hospitals or mortality after discharge were not collected. LOS of ≥4 days was reduced by 15.1% (Table 2). This timeframe was selected for analysis as it was the lowest median value between groups though LOS ≥3 days was found to be more significant on multivariate analysis (AOR 0.33; 95% CI 0.18–0.59; *P* < 0.000). Analysis of this timeframe within specific subgroups may provide added insight into changes in patient outcomes. Overall the median units of blood transfused decreased which took into account hemoglobin levels, GBS score and admissions (Table 4). Specific patient factors including type of comorbid illnesses rather than number, and the specific time of transfusion i.e. before or after endoscopic intervention were not considered and could influence this outcome.

## Conclusion

Following protocol changes at Northern hospital for UGIB management there was an increase in early endoscopy with improvements in rebleeding, LOS ≥4 days and units of blood transfused. While time to endoscopy decreased overall within 24 and 12 h room for improvement remains within the high‐risk subgroups. These findings demonstrate improved outcomes through the streamlining of patient care under a single, dedicated Gastroenterology unit which aided in the overall implementation of revised treatment targets in UGIB management at Northern Hospital. While the prevalence of UGIB continues to decrease, there is an ongoing need for specialized and expedient acute care of these high‐risk patients.
